# Absence of Antiretroviral Therapy and Other Risk Factors for Morbidity and Mortality in Malaysian Compulsory Drug Detention and Rehabilitation Centers

**DOI:** 10.1371/journal.pone.0044249

**Published:** 2012-09-18

**Authors:** Jeannia J. Fu, Alexander R. Bazazi, Frederick L. Altice, Mahmood N. Mohamed, Adeeba Kamarulzaman

**Affiliations:** 1 Yale School of Medicine, Department of Internal Medicine, Section of Infectious Diseases, AIDS Program, New Haven, Connecticut, United States of America; 2 Yale School of Public Health, Division of Epidemiology of Microbial Diseases, New Haven, Connecticut, United States of America; 3 Centre of Excellence for Research in AIDS (CERiA), University Malaya Medical Centre, Kuala Lumpur, Malaysia; 4 Universiti Utara Malaysia, Sintok, Kedah, Malaysia; Beijing Institute of Microbiology and Epidemiology, China

## Abstract

**Background:**

Throughout Asia, people who use drugs are confined in facilities referred to as compulsory drug detention and rehabilitation centers. The limited transparency and accessibility of these centers has posed a significant challenge to evaluating detainees and detention conditions directly. Despite HIV being highly prevalent in this type of confined setting, direct evaluation of detainees with HIV and their access to medical care has yet to be reported in the literature.

**Methods:**

We evaluated the health status of 100 adult male detainees with HIV and their access to medical care in the two largest Malaysian compulsory drug detention and rehabilitation centers holding HIV-infected individuals.

**Results:**

Approximately 80% of all detainees with HIV were surveyed in each detention center. Most participants reported multiple untreated medical conditions. None reported being able to access antiretroviral therapy during detention and only 9% reported receiving any HIV-related clinical assessment or care. Nearly a quarter screened positive for symptoms indicative of active tuberculosis, yet none reported having been evaluated for tuberculosis. Although 95% of participants met criteria for opioid dependence prior to detention, none reported being able to access opioid substitution therapy during detention, with 86% reporting current cravings for opioids and 87% anticipating relapsing to drug use after release. Fourteen percent of participants reported suicidal ideation over the previous two weeks.

**Conclusion:**

We identified a lack of access to antiretroviral therapy in two of the six compulsory drug detention and rehabilitation centers in Malaysia designated to hold HIV-infected individuals and found significant, unmet health needs among detainees with HIV. Individuals confined under such conditions are placed at considerably high risk for morbidity and mortality. Our findings underscore the urgent need for evidence-based drug policies that respect the rights of people who use drugs and seek to improve, rather than undermine, their health.

## Introduction

Throughout Asia, people who use drugs are confined and forced to undergo drug rehabilitation in specialized facilities referred to as compulsory drug detention and rehabilitation centers [1], [2]. These centers are operated by government agencies, although the specific supervising body differs by country [3]. In Malaysia, they are operated by the National Anti-Drug Agency (AADK). Despite their intended purpose of drug rehabilitation, these centers are custodial and managed primarily by military or security personnel [3], [4]. Over the last decade, compulsory drug detention and rehabilitation centers have grown exponentially throughout East and Southeast Asia [4] and currently detain over an estimated 400,000 individuals each year [5]. In 2010, an estimated 6,658 individuals were detained across Malaysia's 28 compulsory drug rehabilitation centers.

Grounds for detention in these centers range from having a positive urine drug screen to being suspected of illicit drug use [3], [6]. Individuals are routinely denied due process and, in some cases, may be detained indefinitely [3], [6]–[8]. Under Malaysia's drug control laws, any individual with a positive urine screening for substances classified as illicit by the Dangerous Drug Act (1952) and the Drug Dependence (Treatment and Rehabilitation) Act (1983) and deemed to be drug-dependent by a government medical officer can be mandated to two years of detention and two years of community supervision following release [9]. Evidence-based therapies for substance use disorders, such as opioid substitution therapy, are typically not provided during detention. Instead, compulsory abstinence, military drills, and other approaches with unproven efficacy are utilized as rehabilitation measures [3], [7], [10]. In some countries, detainees may also be subjected to forced labor and physical abuse [3], [4], [7], [10]. Given these practices, drug-related outcomes are often poor after release, with an estimated 70–90% of those leaving Malaysian compulsory drug detention and rehabilitation centers relapsing to drug use within one year of release [11] and similarly high relapse rates reported across Asia [3], [4].

HIV prevalence is reported to be extremely high in these centers [12], [13]. In Malaysia, where people who inject drugs account for two-thirds of all HIV infections [11], HIV prevalence in compulsory drug detention and rehabilitation centers is estimated to be 10%, nearly two-fold higher than in prisons and 20-fold higher than in the community [14]. Less is known, however, about the provision of HIV treatment and care for detainees. Across Asia, human rights agencies have repeatedly identified limited to no access to antiretroviral therapy in compulsory drug detention and rehabilitation centers based on interviews with former detainees and personnel from non-governmental organizations, and different studies have reported poor, non-HIV related health outcomes among both current and former detainees [2]–[4], [7], [8], [10], [15]–[18].

Despite HIV being highly prevalent in this type of confined setting, direct evaluation of detainees with HIV and their access to medical care has yet to be reported in the peer-reviewed literature. The limited transparency and accessibility of these centers has challenged efforts to evaluate detainees and detention conditions directly [12], [13], [19], [20]. In Malaysia, first-line antiretroviral therapy is fully subsidized, yet, it remains unknown whether this treatment policy is upheld in compulsory drug detention and rehabilitation centers. Interviewing current detainees would provide much-needed information on their health and well-being and further illuminate the conditions inside these settings. We obtained access to the two largest Malaysian compulsory drug detention and rehabilitation centers holding HIV-infected individuals and evaluated the health status of detainees with HIV and their access to medical care during confinement.

## Methods

### Ethics Statement

The Medical Ethics Committee of the University Malaya Medical Centre approved all aspects of the study and the use of oral informed consent. Oral informed consent was obtained from study participants as the only record linking the participant and the research would have been the consent document and we determined that the principal risk would be the potential harm resulting from any breaches of confidentiality. The oral informed consent process was documented by providing individuals with a form outlining the purpose and procedures of the study and a consent statement that individuals only needed to check ‘yes’ or ‘no’ to. This form was also dated and initialed by study interviewers.

Based on the national drug agency's memorandums of agreement with the Ministry of Health, HIV-infected detainees are placed in one of six compulsory drug detention and rehabilitation centers where HIV treatment and care is designated to be accessible. From July to August 2010, 100 HIV-infected adult males were surveyed in the two largest compulsory drug detention and rehabilitation centers housing HIV-infected detainees. Both sites were male-only facilities (there is only one facility in the country that houses women) and were located in Peninsular Malaysia. One facility was located in Besut, Terengganu in the northeast (387 detainees; 54 detainees were HIV-infected) and the other was located in Muar, Johor in the south (407 detainees; 70 detainees were HIV-infected). In each detention facility, HIV testing is mandatory and detainees with confirmed HIV infection are housed separately, involuntarily disclosing their HIV status both to other detainees and to facility staff.

A 90-minute survey was administered that assessed previous involvement with the criminal justice system and compulsory drug detention and rehabilitation centers, drug use prior to detention, self-reported HIV-related symptoms, and access to medical treatment and care and contained validated clinical assessments for opioid dependence (MINI) [21], addiction severity (DAST-10) [22], depression (PHQ-9) [23], and a tuberculosis (TB) symptom screening algorithm for individuals with HIV infection [24]. The MINI and DAST-10 and the TB symptom screening algorithm have not been validated in Malaysia but have previously been validated in other Southeast Asian countries. The PHQ-9 has been used in previous studies in Malaysia but has not been formally validated. The survey instrument was translated from English to Bahasa Malaysia and back-translated to English using previously described methods [25]. Surveys were piloted in Bahasa Malaysia with former drug users and further adapted for linguistic precision and socio-cultural sensitivity.

Researchers were allowed 10 days at each facility for study recruitment. There were no incentives or disincentives for study participation. All HIV-infected detainees at each facility were eligible and notified of the study by trained research assistants fluent in Bahasa Malaysia who visited the segregation units where HIV-infected individuals were housed to describe the study. Individuals expressing interest were escorted by research assistants to confidential interview rooms and taken through an oral informed consent process. Those who agreed to participate in the study provided oral informed consent and were subsequently recruited into the study and surveyed. Study participation was anonymous and no personal identifiers were collected. After data collection was completed, an educational session on HIV treatment and care was provided to all HIV-infected detainees, irrespective of study participation, and to the counselors assigned to address their needs.

Data were analyzed with SPSS 16 (SPSS, Inc., Chicago IL) using Chi-square and Student *t*-tests. P-values<0.05 were considered statistically significant.

## Results

Mandated duties, including work, counseling, required exercises, and religious sessions did not allow for all eligible and willing subjects to be recruited during the interview periods. By the end of each survey period, however, 44 (81.5%) of all 54 eligible detainees in Besut and 56 (80%) of all 70 eligible detainees in Muar had been surveyed. Of the 107 eligible individuals interested in participating in the study, six were deemed too ill to complete the survey and one declined participation after learning more details about the study.

Participant characteristics are summarized in Table 1. There were no significant differences between participants from the two sites with regard to demographic characteristics, health status and access to care (data not shown). Both detention centers reported relying on external medical facilities, primarily government district hospitals, for acute care of HIV-infected detainees, although it was unclear who makes referrals for detainees to be brought to external facilities for care. Aside from a medical assistant in one facility who was trained to address non-urgent medical issues, comprehensive on-site medical services and routine access to medical care were not available at either facility. Most individuals (91%) surveyed were Malay, 70% reported being from a rural area (*kampung*). Participants reported a mean of 3.0 and 2.3 cumulative detentions in prison and compulsory drug detention and rehabilitation centers, respectively. Most detainees (78%) had been diagnosed with HIV in a confined setting, with 20% reporting being diagnosed during this detention.

**Table 1:** Participant Characteristics (n = 100).*

**Age in years, mean (SD)**	35.0 (6.5)
**Ethnicity**	
Malay	91
Chinese	4
Indian	5
**Married**	23
**Completed high school**	33
**Monthly income below poverty level**	15
**Religion**	
Muslim	92
Other	8
**Place of residence prior to detention**	
Rural	70
Urban or suburban	30
**Unstable housing prior to detention**	11
**Detention history**	
Jail incarcerations, mean (SD)	7.6 (5.8)
Prison incarcerations, mean (SD)	3.0 (2.9)
Lifetime number of months incarcerated in a jail or prison, mean (SD)	35.6 (44)
Detentions in compulsory drug detention and rehabilitation facilities, mean (SD)	2.3 (1.7)
Lifetime number of months detained in a compulsory drug detention and rehabilitation facility, mean (SD)	22.0 (21.1)
Age at first incarceration in jail or prison, mean (SD)	27.6 (7.1)
Age at first detention in a compulsory drug detention and rehabilitation facility, mean (SD)	21.9 (5.7)
**Months since entry into the compulsory drug detention and rehabilitation facility, mean (SD)**	7.5 (3.2)
**Location of HIV diagnosis**	
Compulsory drug detention and rehabilitation facility	41
Jail or prison	40
Hospital or ambulatory clinic	19
**Received HIV diagnosis during current detention**	20
**Never been HIV tested prior to diagnosis**	59
**Months since HIV diagnosis, mean (SD)**	64.8 (55.7)

* Where mean (SD) is not indicated, results presented represent both n and % as 100 participants answered all questions resulting in no missing data.

Substance use disorders were highly prevalent among participants, with 95% meeting DSM-IV criteria for opioid dependence prior to detention and 93% reporting substantial or high addiction severity prior to detention. None reported being offered opioid substitution therapy during confinement. Table 2 describes participants' pre-detention drug use. Heroin was the most commonly cited (94%) drug of choice. However, daily use of multiple substances in the 30 days prior to detention was reported by 40% of participants. Drug injection in the 30 days prior to detention was reported by 95% of subjects, with 65% reporting daily heroin injection and 22% reporting daily injection of multiple substances. Despite being detained for a mean of 7.5 months (Table 1), 86% of participants reported having current cravings for opioids and 58% reported current cravings for methamphetamines. Eighty-seven percent of participants reported anticipating relapsing to drug use after release. Prior experience with medication-assisted treatment was limited, with only 24% reporting previous treatment with methadone and 15% with buprenorphine.

**Table 2:** Drug Use Prior to Detention (n = 100).

	Lifetime use	Primary drug of choice	Drug charge resulting in detention	Any use in 30 days prior to detention	Any injection in 30 days prior to detention	Daily use in 30 days prior to detention	Daily injection in 30 days prior to detention
**Alcohol**	92	0	0	20	N/A	4	N/A
**Heroin**	99	94	96	98	81	82	65
**Methadone**	44	0	0	16	2	7	0
**Buprenorphine or buprenorphine/naloxone**	47	1	0	14	10	5	4
**Benzodiazepines**	55	0	1	20	17	4	3
**Amphetamine-type stimulants**	89	1	1	64	28	21	14
**Ketamine**	21	0	0	5	1	0	0
**Cannabis**	81	2	1	16	N/A	8	N/A
**Multiple substances**	91	N/A	N/A	76	38	40	22

The mean time since HIV diagnosis among participants was 5.4 years, yet none reported being able to access antiretroviral therapy during detention and only 9% reported receiving any HIV-related clinical assessment or care. Sixty-nine percent of participants believed their HIV infection had become symptomatic. Additionally, 74% of participants reported that since their diagnosis, they had not received any further HIV-related clinical assessment or care. While 97% of participants reported wanting to know their current CD4 lymphocyte count, only 34% had ever been CD4 tested and only 18% had ever received a CD4 test result. Of those who had ever received a CD4 test result (n = 18), the median CD4 count was 315 cells/mL (range 15–1025 cells/mL). However, 61% of these reported CD4 test results were from over two years ago. Although 14% of participants had previously been recommended by a medical professional to initiate antiretroviral therapy, only four participants had ever done so. Three of these four participants reported they were on antiretroviral therapy in the 30 days prior to detention and were forced to discontinue treatment due to its unavailability in the detention facility. The vast majority (87%) of participants reported wanting to access HIV care after their release.

Twenty-three percent of participants screened positive for symptoms indicative of active tuberculosis based on a screening algorithm (sensitivity 93%; specificity 36%) using three indicators, prolonged cough (65%), fever (56%) and night sweats (30%) for ≥3 weeks in the preceding month [24]. However, none of these individuals reported having received any assessment for tuberculosis during confinement. Fourteen percent of participants reported suicidal ideation over the previous two weeks.

## Discussion

We identified a lack of access to antiretroviral therapy in two of the six Malaysian compulsory drug detention and rehabilitation centers holding HIV-infected individuals and found significant, unmet health needs among detainees with HIV. Most of our participants reported multiple, untreated and potentially life-threatening medical conditions. Despite antiretroviral therapy being fully government-subsidized, none of our participants reported being able to access antiretroviral therapy during detention, including those who were on therapy prior to detention. Few participants reported receiving any HIV-related clinical assessment or care, and none of the 23 individuals screening positive for symptoms indicative of active tuberculosis reported being clinically evaluated. A quarter of participants reported recent suicidal ideation and nearly all participants met criteria for opioid dependence prior to detention and anticipated relapsing to drug use after release. It is clear from these findings that individuals are being confined under conditions that place them at considerably high risk for morbidity and mortality, both during detention and after release.

We surveyed only 80% of all eligible individuals, potentially introducing selection bias. However, even if those who had not been surveyed reported better outcomes, our findings would remain concerning in both magnitude and severity. Our study also only evaluated compulsory drug detention and rehabilitation centers in Malaysia and thus is limited in its generalizability. Despite these important limitations, our findings provide further evidence of life-threatening conditions in this type of confined setting. Increasingly, many international agencies and actors, including UNAIDS, UNODC, UNDP, UNICEF, the WHO, the Global Fund to Fight AIDS, Tuberculosis and Malaria, and the UN High Commissioner for Human Rights have called for the closing of compulsory drug rehabilitation and detention centers and the implementation of evidence-based drug policies that recognize the rights of people who use drugs [26].

Beginning in 2010, the national drug agency in Malaysia began closing compulsory drug detention and rehabilitation centers throughout the country and converting the physical spaces into voluntary drug treatment centers. These new voluntary treatment centers offer access to fully-subsidized methadone maintenance therapy and other health and social services [27]. Thus far, 10 of the 28 national detention centers have been closed. Examination of this ongoing policy transition is needed and findings related to its impact and cost-effectiveness may provide further support for this transition in Malaysia and may potentially galvanize similar policy change in other countries. In particular, the newly opened voluntary treatment centers and their acceptability among people who use drugs must be closely evaluated given their former status as detention centers. Evaluation efforts can identify potential strengths of the new system as well as areas in which service delivery and accessibility can be improved to meet the needs of those seeking care.

Access to opioid substitution therapy, an essential medicine and key HIV prevention modality, will be vastly expanded if the national drug agency completes this transition to a voluntary, treatment-based system. Conversely, even with this policy shift, people who use drugs will continue to confront significant barriers to accessing treatment and care for HIV. While none of the participants in our study were able to access antiretroviral therapy during confinement, their access in the community was similarly limited. Despite first-line antiretroviral therapy being fully subsidized, only four participants reported ever being on antiretroviral therapy, only a quarter of participants reported receiving any further HIV-related clinical assessment or care since their diagnosis, and only 18 participants reported ever receiving a CD4 test result. In Malaysia as well as across Asia, people who use drugs face especially limited access to HIV treatment and care due to stigma, discrimination and criminalization [2], [28]–[30]. These treatment barriers have serious implications for their health, well-being and survival but, thus far, have not garnered the requisite attention or mobilization. Given the additional prevention benefit of antiretroviral therapy, these treatment barriers also significantly hamper HIV prevention efforts and overall efforts to address the rapidly growing regional epidemic.

Targeted efforts to improve access to HIV treatment and care for this population have yet to be engaged in Malaysia and are urgently needed. These efforts should be rooted in community-based strategies, such as engaging outreach workers in needle and syringe exchange programs in HIV testing and linkage to care and actively integrating and co-locating treatment services for HIV, TB, hepatitis C and substance use disorders in drug treatment centers. Needle and syringe exchange programs and drug treatment centers are the primary points of access to people who use drugs throughout the country, but these settings have yet to be utilized in delivering other critical health services to this population, such as voluntary HIV testing and provision of or linkage to HIV treatment and care. While HIV testing typically serves as the gateway for entry into care, the vast majority of our participants were first diagnosed with HIV in a prison or compulsory drug detention and rehabilitation facility. Confined settings where HIV testing is mandatory, individuals testing positive are segregated, and treatment and care are largely unavailable [31] should not be the primary contexts in which people who use drugs are being tested for HIV. The rest of our participants (20%) reported receiving their first positive HIV test result in a hospital or ambulatory clinic, with no other settings reported. Given the marginalization of people who use drugs, voluntary HIV testing venues outside of traditional healthcare settings would likely be far more effective in reaching this population. Our findings highlight the importance of expanding community-based, voluntary HIV testing programs that are equipped to both diagnose and promote entry into care for this marginalized group.

The extremely low levels of access to HIV treatment and care in the community reported by our participants may also be related to the fact the majority were from rural areas, where there is less infrastructure for diagnosing and treating individuals with HIV/AIDS and greater stigmatization of drug use and HIV. Antiretroviral therapy may be subsidized in Malaysia but the actual provision of treatment and care ultimately rests upon health care providers. Efforts to increase access to treatment for people who use drugs must engage the high levels of stigmatization of HIV and drug use among Malaysian providers and actively address misperceptions and discriminatory practices that compromise treatment and care.

## Conclusions

Detention and forced rehabilitation continues to be the cornerstone of national drug policy in many Asian countries. The profoundly negative impact of this punitive approach on people who use drugs, their health and the health and well-being of communities and society at large is more visible than ever before and should be considered a health and human rights emergency. Findings from this study bolster the ongoing closure of compulsory drug detention and rehabilitation centers in Malaysia and underscore the urgent need for evidence-based drug policies that respect the rights of people who use drugs and seek to improve, rather than undermine, their health.
