# Correction: Absence of Antiretroviral Therapy and Other Risk Factors for Morbidity and Mortality in Malaysian Compulsory Drug Detention and Rehabilitation Centers

**DOI:** 10.1371/annotation/d8cc1b96-93f6-40ce-88eb-c52acebaad05

**Published:** 2013-02-01

**Authors:** Jeannia J. Fu, Alexander R. Bazazi, Frederick L. Altice, Mahmood N. Mohamed, Adeeba Kamarulzaman

The TB screening algorithm used in the study to identify individuals with symptoms indicative of active tuberculosis is actually based on reporting ANY of three primary symptoms, not ALL three primary symptoms, thus the number of individuals with symptoms indicative of active tuberculosis should have been 79%, not 23%. This finding appears once in the Abstract, once in the Results, and once in the Discussion.

